# A Giant Eyelid Molluscum Contagiosum Revealing an HIV Infection: A Case Report and Literature Review

**DOI:** 10.7759/cureus.41187

**Published:** 2023-06-30

**Authors:** Amer Alghamdi, Yousef S Alghamdi, Hany Hanafi, Mohamed M Karami, Waleed Khayyat, Reem M Hersi, Nada K Naaman

**Affiliations:** 1 Department of Ophthalmology, College of Medicine, King Saud University, Riyadh, SAU; 2 Department of Ophthalmology, East Jeddah Hospital, Jeddah, SAU; 3 Department of Ophthalmology, East Jeddah General Hospital, Jeddah, SAU; 4 Department of Clinical Physiology, Faculty of Medicine, King Abdulaziz University, Jeddah, SAU; 5 Department of Ophthalmology, King Khaled Eye Specialist Hospital, Riyadh, SAU; 6 College of Medicine, King Saud Bin Abdulaziz University for Health Sciences, Jeddah, SAU

**Keywords:** poxvirus, molluscum contagiosum, human immunodeficiency virus infection, eyelid, case report

## Abstract

Molluscum contagiosum (MC) is a common benign cutaneous viral infection. It can affect any part of the skin with a high propensity for facial skin, especially in human immunodeficiency virus (HIV) patients with low CD4 count. We report a case of a 16-year-old female patient who presented with a giant isolated right upper eyelid MC lesion that served as the first clinical indicator of her HIV infection and acquired immunodeficiency syndrome (AIDS). A final diagnosis of MC was made based on the history, clinical findings, and histopathological examination. Moreover, due to its vital location, large size, and atypical presentation, a surgical excision by simple unroofing and curettage was performed under local anesthesia to speed recovery, prevent corneal complications, and reduce transmission. Her follow-up visits showed satisfactory clinical and cosmetic outcomes. Patients presenting with giant atypical eyelid lesions must be thoroughly investigated for immunosuppressive states, especially HIV infection. MC can have atypical presentations in HIV patients. To our knowledge, this is one of a few cases in the literature reporting a giant isolated eyelid MC lesion leading to a diagnosis of HIV infection with AIDS.

## Introduction

Molluscum contagiosum (MC) is a common benign cutaneous viral infection caused by poxvirus. It manifests as dome-shaped, umbilicated flesh-colored papules that are mostly found on mucus membranes or skin. MC is commonly observed in immunosuppressed patients, particularly those with human immunodeficiency virus (HIV) infection [1]. In some individuals, MC may serve as the first clinical indicator of an HIV infection [2]. Few cases of giant MC have been reported, mostly in HIV-positive patients [2]. It is uncommon to have widespread MC lesions in the body [3] or lesions in certain body parts especially the eyelids [4]. This report describes a case of a 16-year-old female patient who presented with an unusually large right eyelid MC lesion, which after subsequent investigations, revealed a concurrent HIV infection, leading to acquired immunodeficiency syndrome (AIDS). After surgical removal, the cosmetic and functional outcomes were satisfactory. This work has been reported in line with the SCARE (Surgical CAse REport) criteria [5]. The CARE (CAse REport) checklist has been completed by the authors for this case report and is attached as supplementary material.

## Case presentation

A 16-year-old Saudi female presented to the ophthalmology clinic with a chief complaint of an enlarging lesion in the right upper eyelid for eight months, obscuring her visual field in the right eye. The patient denied any other systemic symptoms or similar lesions in other body sites. Her family history was unremarkable. She had no known past medical or surgical history, including no diagnosis of immunodeficiency. She did not receive hepatitis B virus (HBV) or human papillomavirus (HPV) vaccinations. She lived in a low-socioeconomic, humid, and crowded environment and occasionally smoked but has no history of alcohol consumption or illicit drug use. The patient refused to consent to the disclosure of any additional information that could reveal the route of infection transmission.

**Figure 1:**
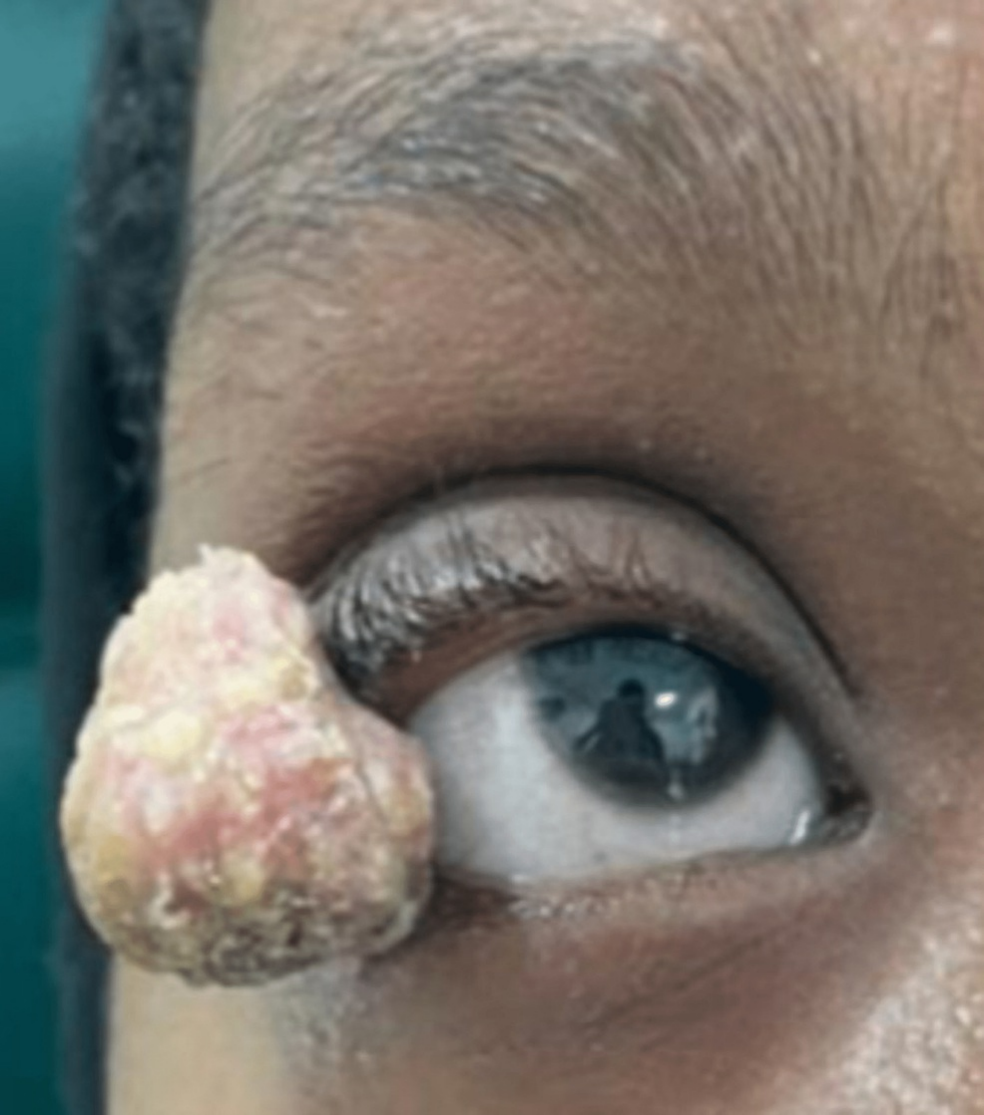
Large right upper eyelid lesion measuring 1.9 x 1.5 x 1.2 cm.

On examination, there was a single large exophytic grey-tanned, flesh-colored tumor over the lateral aspect of the right upper eyelid with an overlying skin crust, without signs of a secondary bacterial infection (Figure 1).

On palpation, the lesion was soft and friable in consistency, non-tender, and without inflammatory signs or abnormal discharge. On ocular examination, her visual acuity was 20/20 without correction in both eyes with intact ocular movements. On confrontation, there was a temporal visual field defect in the right eye that resolved upon elevation of the upper lid. The anterior and posterior segments of both eyes were within normal limits. Also, systemic physical examination was unremarkable except for multiple genital warts. 

As part of the blood tests to investigate this atypical giant lesion, an HIV test was performed which revealed a positive status. The patient then was referred to the infectious diseases (ID) department for further evaluation of the HIV infection and initiation of the appropriate management protocol. Her laboratory tests at the time of the diagnosis showed a high level of HIV viral load (42020 copies/ml), a CD4 count of 28 cell/μl, and a CD 8+ count of 921 cell/μl. Infectious disease screening tests for syphilis, toxoplasmosis serology, HBV, and hepatitis C virus (HCV) were negative. Additionally, her hemoglobin level, glycated hemoglobin (HbA1c) level, complete blood count (CBC), platelets count, liver function tests (LFT), and renal function tests were all within normal range. 

**Figure 2:**
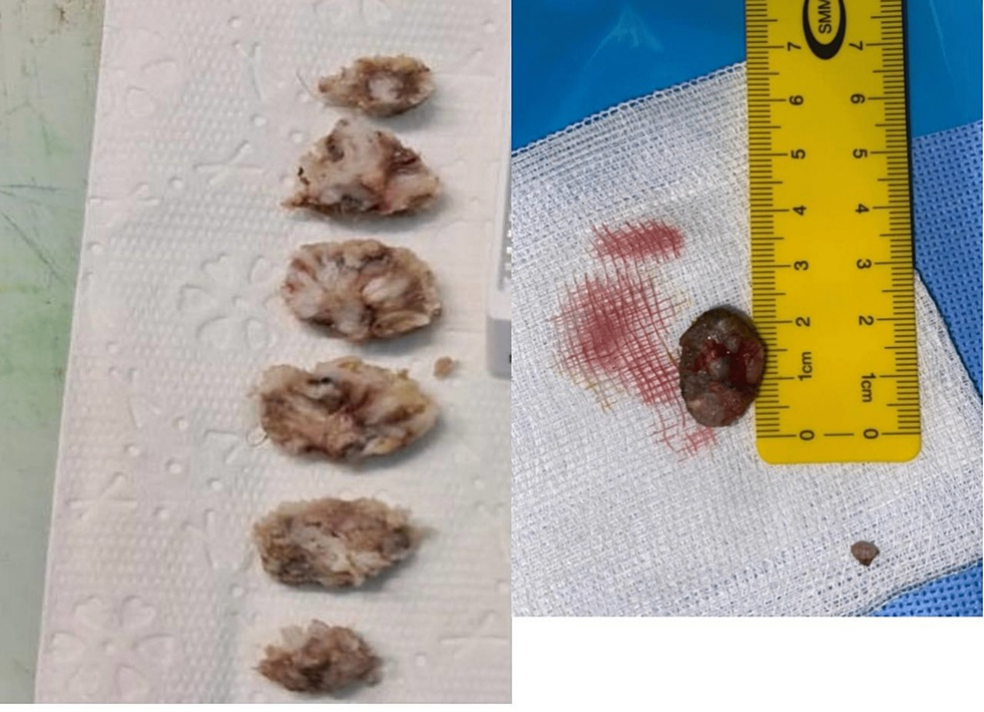
The molluscum contagiosum (MC) lesion post surgical excision.

Due to the cosmetic and functional compromise, a surgical excision by simple unroofing and curettage under local anesthesia was offered to the patient. An eyelid reconstruction was not indicated due to the pedunculated nature of the lesion and a base occupying only a small area of the eyelid. After surgical excision, the lesion was measured to be 1.9 x 1.5 x 1.2 cm in size, and underneath it, a separate small lesion (0.4 x 0.3 x 0.3 cm) of similar features was discovered (Figure 2). Histopathological reports of both lesions described large suprabasilar cells and eosinophilic intracytoplasmic inclusions (molluscum bodies) with the presence of small eccentric nuclei. Although the epithelium was acanthotic, it was mature to the surface. Also, the superficial dermis showed dense chronic inflammatory cells including lymphocytes, eosinophils, and plasma cells. The specimen was negative for malignancy.

**Figure 3:**
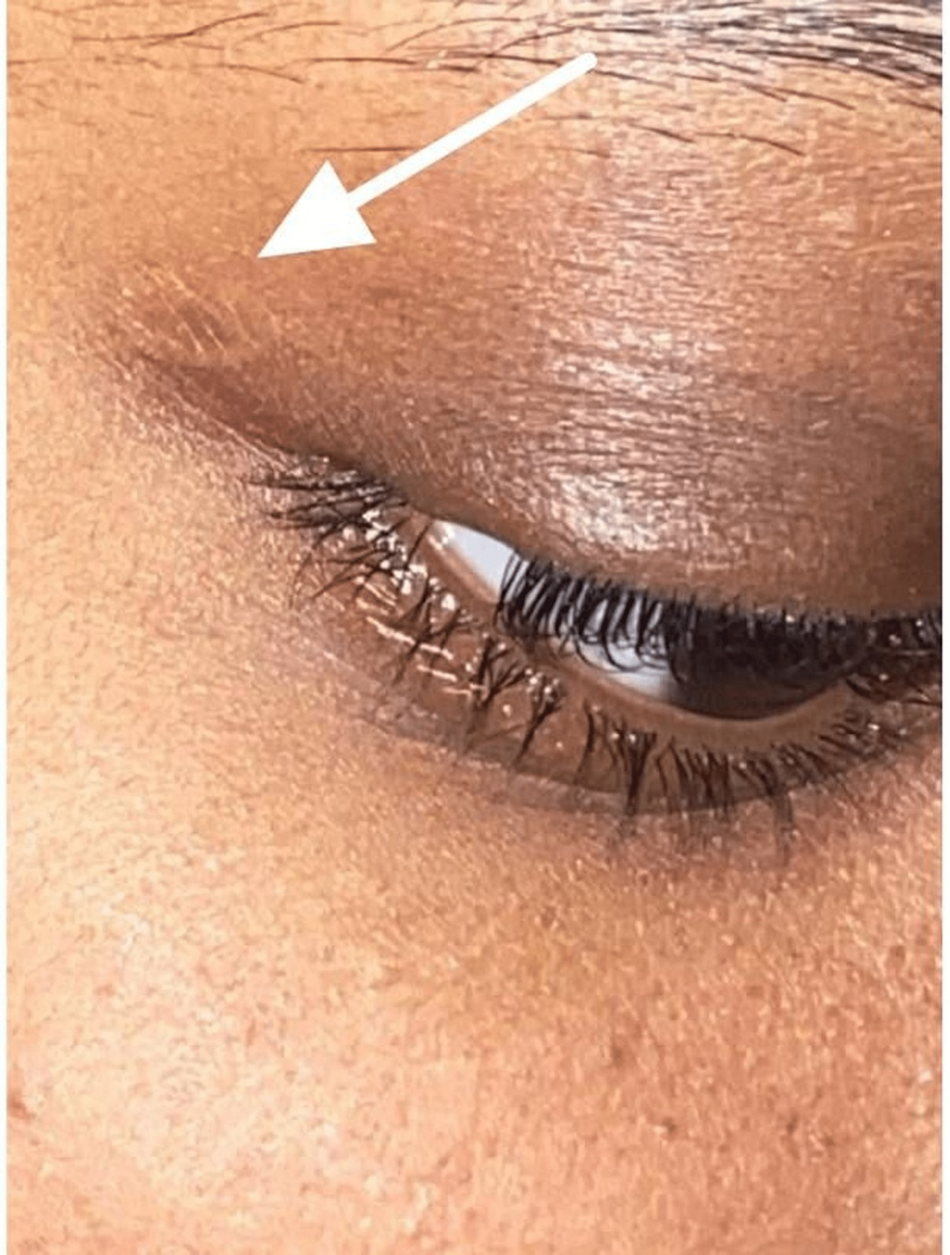
Right upper lid following surgical excision of the lesion.

A final diagnosis of MC was made based on the history, clinical findings, and histopathological examination. The eyelid had a satisfactory cosmetic outcome and the patient was instructed on good hygiene practices to avoid transmission of the virus (Figure 3). 

The ID department in the hospital and the public health office were notified of the case. Her ID physician started her on tenofovir/emtricitabine (Descovy), dolutegravir (Tivicay), and added a sulfamethoxazole on her third-month follow-up visit as the HIV status was worsening again. Regarding the MC lesion, the clinical and cosmetic outcomes were satisfactory without a recurrence at six months post the excision. 

## Discussion

MC is a common benign cutaneous viral infection. It is a member of the poxviridae family, presenting as umbilicated flesh-colored papules. These dome-shaped lesions are mostly found on the skin or mucus membranes. Although their sizes range from 1 to 5 mm in diameter, few as large as 1.5 cm in diameter have been found, mostly in HIV patients [2,6,7]. Lesions larger than 0.5 cm are defined as giant lesions. 

MC is commonly observed in children, sexually active adults, and immunosuppressed patients [6,8]. The onset of HIV-associated MC ranges from infancy and early childhood [9] to middle-aged adults [10]. Additionally, in some individuals, MC may serve as the first clinical indicator of HIV infection, like in our case [2,10]. The local public health office was notified of our case to further investigate the source of the infection. Given the cultural and religious context in Saudi Arabia, the patient’s wish to keep information pertaining to the source of infection private is not unexpected. HIV stigma has long been known as a major barrier to testing and effective patient management [11]. The stigma extends beyond healthcare access to involve negative perceptions and attitudes toward HIV patients by their partners, families, and communities [12,13]. The prevalence of HIV remains relatively low in Saudi Arabia, owing to the negative association of Islamic religious affiliation with HIV transmission [14,15]. However, case notifications among Saudi nationals have been reportedly increasing, possibly due to urbanization and a relative decrease in disease stigmatization [14].

MC is most likely underreported due to its benign and often self-limiting nature. Although the worldwide incidence was estimated to be between 2% and 8% [16], recent studies suggested that the true incidence is much higher [17]. Although these lesions are generally asymptomatic as in our patient, they may also become painful or pruritic. MC lesions usually resolve within 18 months but may persist for prolonged periods, especially in those with weakened immune systems [18]. Several cases have been reported before in the literature. Table 1 summarizes the cases reported previously of isolated eyelid MC lesions in HIV patients [2,4,9,19-22]. 

**Table 1:** Literature review of isolated eyelid MC lesions as a presenting sign of HIV. *MC: molluscum con­tagiosum

Table 1: Literature Review of Isolated Eyelid MC Lesions as a Presenting Sign of HIV				
Author	Presentation	Diagnosis	Treatment	Prognosis
Achdiat et al. 2021 [19]	1st case: Age: 36-year-old Gender: male Complaint: multiple pruritic papules for seven months. Location: upper and lower eye lids on both eyes. Size: started as pin-point and increased to 1 cm. Associated symptoms: eye redness and discharge. Known HIV: Yes CD4 Count: 31 cells/μL Previous MC infection: No	Biopsy: Henderson–Paterson bodies. Diagnosis: Giant MC.	Treatment: 20% KOH solution. Side effects: red eye and burning sensation.	Improvement was observed 4 weeks after the initiation of the treatment with complete resolution of the papules.
Achdiat et al. 2021 [19]	2nd case: Age: 26-year-old Gender: Female Complaint: Multiple itchy papules which multiplied in size for six months. Location: upper and lower left eyelid. Size: started as pin-size and increased to 1 cm. Known HIV: Yes CD4 Count: 46 cells/μL Previous MC infection: No	Biopsy: Henderson–Paterson bodies. Diagnosis: Giant MC.	Treatment: 20% KOH solution. Side effects: erythema and edema of the eyelid.	Lesions appeared smaller after three weeks and completely cleared after four weeks of therapy.
Nair et al. 2016 [20]	Age: 6-year-old. Gender: Male Complaint: multiple pedunculated nodules with central umblication for three months. Location: eyelids of both eyes. Size: N/A Known HIV: No Previous MC infection: No	Biopsy: eosinophilic intracytoplasmic inclusion bodies. Diagnosis: MC and f HIV infection. CD4 count: 124/ml3.	Treatment: High active antiretroviral therapy. Side effects: none	Full recovery without recurrence.
Massa et al. 2013 [4]	Age: 41-year-old Gender: female Complaint: umbilicated pearly papules for two months. Location: right upper eyelid. Size: N/A Known HIV: Yes CD4 Count: 22 cells/mm³ Previous MC infection: Yes	Biopsy: Henderson- Patterson bodies. Diagnosis: MC.	Treatment: Three sessions of 70% trichloroacetic acid after curettage. Side effects: none.	Patient exhibited good response to treatment with regular follow-up visits.
Averbuch et al. 2009 [9]	Age: 6-year-old Gender: Male Complaint: non-tendermultiple shiny nodules Location: upper and lower eyelids of both eyes. Size: 1.5 cm. Associated symptoms: hepatosplenomegaly and lymphadenopathy. Known HIV: Yes CD4 Count: 42 cells/mm3 Previous MC infection: No	Diagnosis: MC.	Treatment: Nevirapine, zidovudine and lamivudine. In addition to trimethoprim-sulfamethoxazole as prophylaxis. Side effects: none.	Complete resolution of the eyelids and facial lesions with residual scarring after 6 months of treatment.
Biswas et al. 1997 [21]	Age: 4-year-old Gender: male Complaint: painful swelling of the right eyelid with multiple nodular lesions for the last 6 months. Location: Right eyelid Size: 1-15 mm Associated symptoms: Purulent discharge from the lesions. Known HIV: Yes CD4+ Count: N/A Previous MC infection: No	Biopsy: round and oval eosinophilic intracytoplasmic inclusion bodies. Diagnosis: MC infected with Staphylococcus aureus, alpha-haemolytic streptococci, Corynebacterium xerosis, Candida lipolytica, and Bacteriodes intermedius.	Treatment: Cloxacillin and ciprofloxacin eye ointment. Side effects: none.	Lid edema subsided two weeks after therapy.
Albini and Rao 2003 [22]	Age: 46-year-old Gender: female Complaint: shiny, centrally umbilicated papule for one month. Location: left lower eyelid. Size: 2mm. Associated symptoms: burning, itching, and tearing. Known HIV: Yes CD4 Count: 435610 /l. Previous MC infection: No	Biopsy: Intranuclear and intracytoplasmic inclusion bodies. Diagnosis: MC.	Treatment: Excision.	No recurrence
Leahey et al. 1997 [2]	Age: 34-year-old Gender: male Complaint: multiple lesions for three months. Location: all four eyelids. Size: N/A Known HIV: No CD4 Count: N/A Previous MC infection: No	Biopsy: large intracytoplasmic, faintly basophilic inclusions. Diagnosis: MC and HIV. CD4 count was 22 cells/mm.3	Treatment: Excision	Patient died 10 months after initial presentation.

In HIV patients, single or multiple lesions can be found on the face, neck, or genitals, and are rarely widespread in the body [3] or the eyelids [4]. In addition, HIV patients may exhibit atypical presentations such as a giant MC [23], as a cutaneous correlate of a deteriorating cellular immunity, a late manifestation of HIV infection [24]. Diagnosis is usually established clinically, however, pathologic examination through a skin biopsy is important sometimes, especially in immunocompromised patients who may present with other opportunistic infections or malignancies that mimic MC [25,26].

MC is not always treated as it might resolve spontaneously within approximately 18 months in healthy subjects. Prompt removal of the lesions - using locally destructive therapy (curettage, cryotherapy, and/or laser) or surgical excision has been described in the medical literature [8]. Other modalities include CO2 laser, imiquimod, trichloroacetic acid, and/or antiviral medication [27]. These treatments are indicated in patients who have persistent lesions, are concerned about cosmesis, and are at risk of inadvertent autoinoculation, particularly in children. Unfortunately, HIV patients often present with lesions that are resistant to standard therapies and may benefit from combined modalities [28]. 

## Conclusions

Patients presenting with giant atypical eyelid lesions suspicious for MC should be thoroughly investigated for immunosuppressive states, especially HIV infection. MC can have atypical presentations in HIV patients.
